# Color-Space-Based Visual-MIMO for V2X Communication †

**DOI:** 10.3390/s16040591

**Published:** 2016-04-23

**Authors:** Jai-Eun Kim, Ji-Won Kim, Youngil Park, Ki-Doo Kim

**Affiliations:** Department of Electronic Engineering, Kookmin University, Seongbuk-gu, Seoul 136-702, Korea; eun9477@kookmin.ac.kr (J.-E.K.); kjwon777@kookmin.ac.kr (J.-W.K.); ypark@kookmin.ac.kr (Y.P.)

**Keywords:** visual-MIMO, color independence, multiplexing/diversity, mobile optical networking, V2X

## Abstract

In this paper, we analyze the applicability of color-space-based, color-independent visual-MIMO for V2X. We aim to achieve a visual-MIMO scheme that can maintain the original color and brightness while performing seamless communication. We consider two scenarios of GCM based visual-MIMO for V2X. One is a multipath transmission using visual-MIMO networking and the other is multi-node V2X communication. In the scenario of multipath transmission, we analyze the channel capacity numerically and we illustrate the significance of networking information such as distance, reference color (symbol), and multiplexing-diversity mode transitions. In addition, in the V2X scenario of multiple access, we may achieve the simultaneous multiple access communication without node interferences by dividing the communication area using image processing. Finally, through numerical simulation, we show the superior SER performance of the visual-MIMO scheme compared with LED-PD communication and show the numerical result of the GCM based visual-MIMO channel capacity *versus* distance.

## 1. Introduction

**Figure 1:**
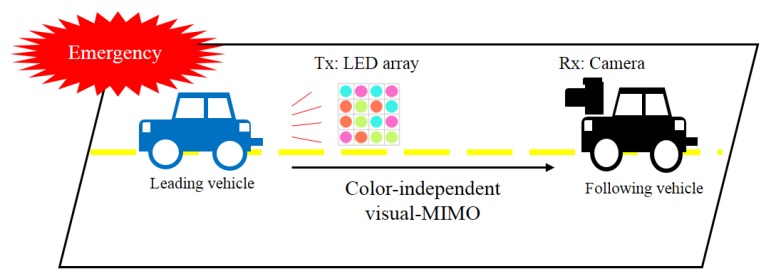
Illustration of the optical V2V communication system.

In [1], it has been argued that it is now becoming feasible to achieve high data rates over larger transmission ranges in mobile optical wireless communications using camera receivers through a concept called ‘visual multiple-input multiple-output (visual-MIMO)’. In this concept, multiple transmit elements of a light emitting array (LEA) are used as transmitters to communicate with the pixels of the camera which act as multiple receiver elements to create the visual-MIMO channel. Using MIMO techniques such as “multiplexing” to send independent streams of bits using the multiple elements of the light transmitter array and recording over a group of camera pixels can further enhance data rates. On the other hand, the system can send the same information on all the transmit elements of the array and use “diversity” combining at the camera to achieve larger transmission range due to the signal-to-noise ratio (SNR) gain [1]. Although the multiplexing and diversity techniques are similar in concept to those in RF MIMO systems, the visual-MIMO channel with very different characteristics attributes certain unique behavior to the MIMO gains in these systems. Applications are not limited to hand-held cameras and electronic displays but also include vehicle-to-vehicle (or road) communication, robot-to-robot communication, and hand-held displays for fixed surveillance cameras. Figure 1 shows an example of the application of visual-MIMO for vehicle-to-everything (V2X) [2,3].

The modifier ‘color-independent’ indicates the independence of the variations in the light color and light intensity. Thus, we may achieve a visual-MIMO scheme that can maintain the original color and brightness while performing seamless communication. In this paper, we analyze the applicability of color-independent visual-MIMO for V2X.

## 2. Experimental Section

### 2.1. Visual-MIMO with Color-Space-Based Modulation

The first color-space-based modulation scheme, termed color shift keying (CSK), was proposed by the IEEE 802.15.7 task group [4,5]. However, CSK is not suitable for communications when target color varies with time because it does not involve a systematic manner of coping with changing target color conditions. Here, target color means the wanted color of the LED lighting. In order to overcome this limitation, another color-space-based modulation scheme, termed as generalized color modulation (GCM), was proposed for color-independent visible light communication (VLC) systems [6,7]. It should be noted that the most distinctive feature and advantage of GCM over the other modulation schemes is color independency. GCM is able to generate any color within a gamut by combining some of the wavelengths or colors. Therefore, through GCM, we may achieve a VLC scheme that can maintain the original color and brightness while performing seamless communication.

**Figure 2:**
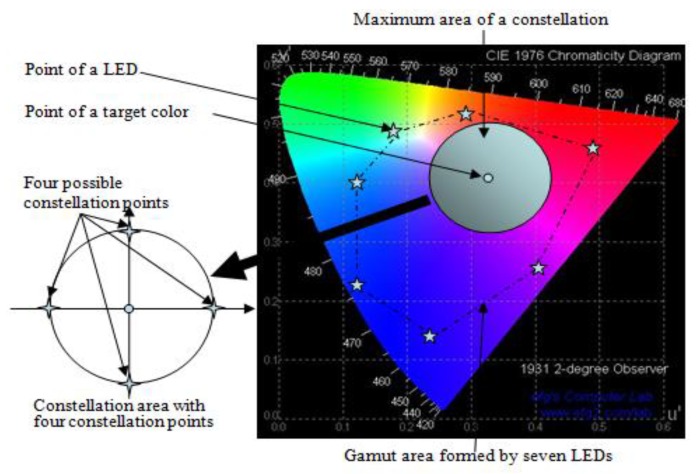
Generation example of a constellation diagram for a target color in CIELUV color space (example uses seven LEDs and two bit data symbols).

GCM constructs a constellation diagram in a light color space to represent data symbols. Each constellation point in a color space represents a corresponding color. The target color, *i.e.*, the color perceivable to human eyes after modulation, is the average of all appropriate constellation points. A simple example of a constellation generation for a target color is illustrated in Figure 2 [7]. Here, constellation points in the color space can be arranged using a similar arrangement to that used in RF circular quadrature amplitude modulation (QAM). Supposing an equiprobable symbol transmission, which is reasonable, due to the compensation and interleaving algorithms, the target color can be obtained as the averaged RGB value along a number of symbols as shown in Equation (1).

(1) $$\left(x_{t},\;y_{t}\right)\;=\;\left(\frac{\sum_{i=1}^{N}x_{i}}{N},\;\frac{\sum_{i=1}^{N}y_{i}}{N}\right)$$

where $\left(x_{t},\;y_{t}\right)$ denotes the position of a target color and $\left(x_{i},\;y_{i}\right)$ denotes the position of the $i_{th}$ symbol. Note that the value of $\left(x_{t},\;y_{t}\right)$ is more close to the true target color when N is increasing in the probability sense.

**Figure 3:**
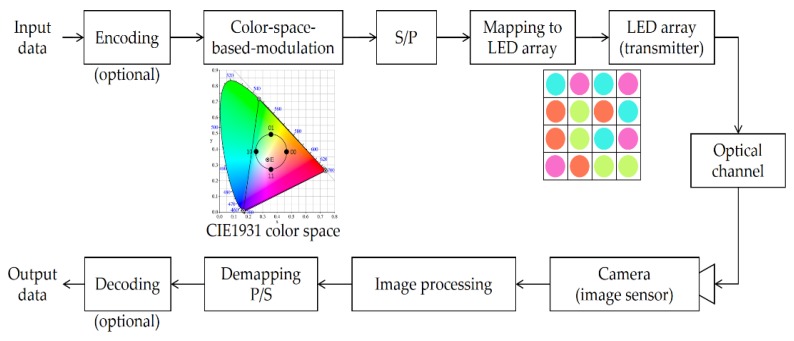
Color-space-based, color-independent visual-MIMO tranceiving procedure using image processing.

Figure 3 shows the block diagram of the proposed color-independent visual-MIMO based on the color space [2]. By incorporating GCM into visual-MIMO, we may obtain a better symbol error rate (SER) performance, higher data rate over a larger transmission range, and most importantly, color independency when compared with conventional light-emitting diode (LED) communication. With this scheme, we can obtain a target color at a time without difficulty by the parallel transmission of different symbols (colors) through the LED array. In this case, we know that N in Equation (1) is the number of total LEDs of the LED array. It is also important to note that we may prevent the burst error occurring during the transition of target color change by transmitting symbols in parallel through LED array of color-independent visual-MIMO transmitter in Figure 3. In [8], we have shown that many errors could occur during the transition of an arbitrary target color to another when the transmitter does not send the target color and the receiver is a photo detector (PD) instead of a camera. Furthermore, the proposed system rapidly and easily adapts to the variations in the target color using image processing.

Generally, the capacity of a color-independent visual-MIMO system depends on the number of bits per symbol, the array size (M × M), and the frame rate of the camera. In our proposed system, the capacity is defined as Equation (2):

(2)  Capacity [bps]=n [bits/symbol] × N [symbols/frame] × F [frames/sec]   

where N = M × M.

### 2.2. Mobile Networking through Visual-MIMO

In contrast to RF wireless channels, the visual-MIMO system will not be subject to multipath fading [9]. Since achievable bitrates of the visual-MIMO system are primarily dependent on receiver perspective, line-of-sight (LOS) availability, and the distance between the transmitter and receiver, our visual-MIMO protocols could benefit from networking information such as distance, angle, inter-symbol-interference (ISI), link bit rates, target color, and reference color (symbol). Such knowledge is useful both at the physical and network layers. If we send the reference color (symbol), we may achieve improvements in the SER performance using noise cancellation. By using the networking information of distance or SNR, we can determine the transition from the multiplexing mode to the diversity mode.

The main challenge facing the deployment of vehicular *ad hoc* networks (VANETs) is the lack of infrastructure. This makes cooperative diversity an ideal physical-layer solution for VANETs. A source node can get help from other nodes by relaying the information message to the destination node. This characteristic of the network allows multipath transmission strategies that are similar in concept to cooperative RF communications, but the former requires less coordination overhead.

**Figure 4:**
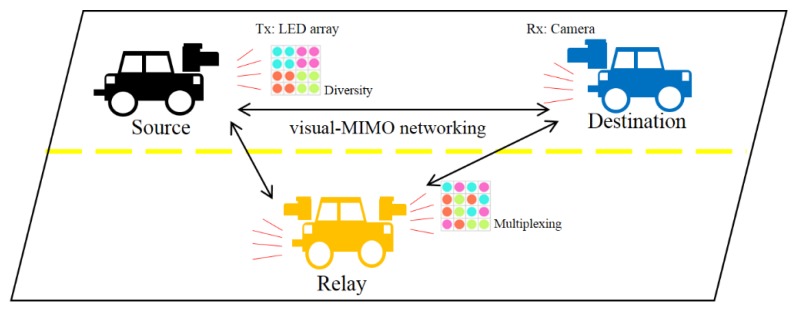
Multipath transmission strategies using geometric information.

Consider a scenario shown in Figure 4 [2,9] involving three nodes, namely a source, a destination, and a potential relay. The relay is positioned between the two other nodes, but remains closer to the source than the destination (without obstructing the LOS between the source and destination). Because shorter distances allow higher multiplexing gain, it is likely that the link capacity between the source and the relay is greater than the other links. The highest throughput can be achieved by simultaneous transmission through the relay and on the direct link. The transmitters can again use networking information to determine the transmission strategies.

**Figure 5:**
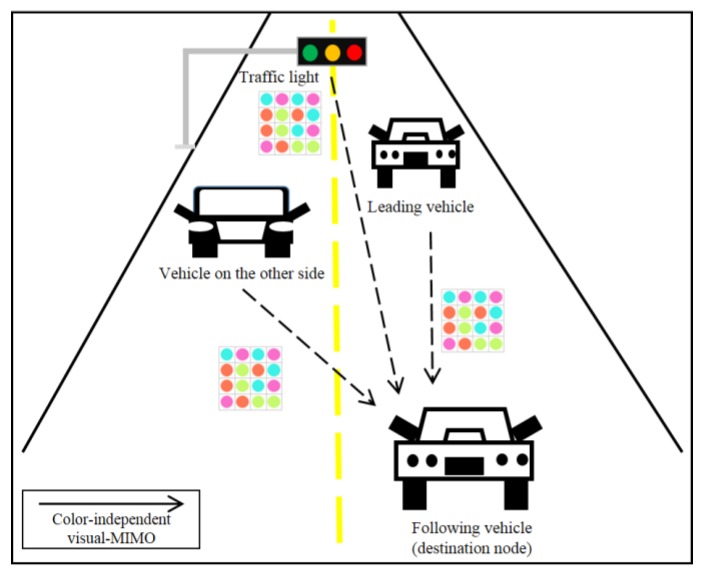
Multi-node V2X communication.

We may also consider another V2X scenario of multiple access in which a destination node can receive the information from multiple source nodes simultaneously. For example, as shown in Figure 5, any driving car needs to communicate with forward vehicles, vehicles driving in the opposite direction and traffic infra-structures such as traffic lights.

In this concept, which we call a visual-MIMO, optical transmissions by multiple transmitter elements are received by an array of photodiode elements (e.g., pixels in a CMOS camera). The image sensor in a camera is essentially an array of photodiodes and the camera lens provides a different narrow field of view for each photodiode. This creates a large number of highly directional receive elements (the camera pixels), which allows reduction of interference and noise and thereby can achieve large ranges, yet still maintain the wide field-of-view necessary for mobile communication [9].

**Figure 6:**
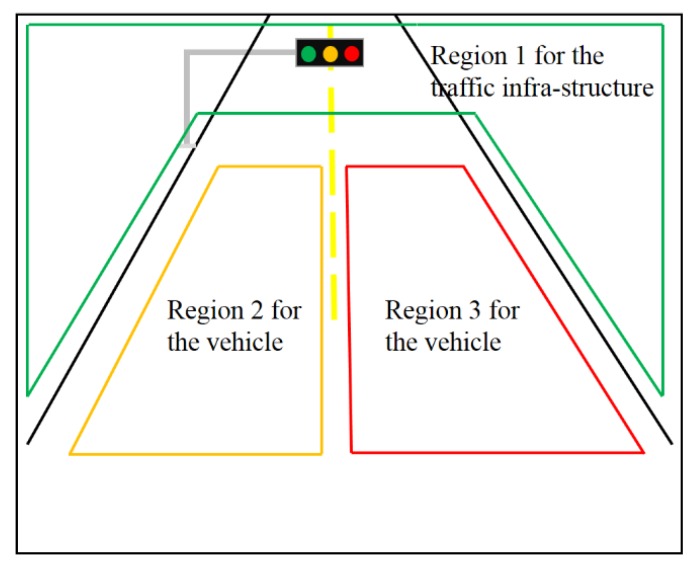
The divided ROI for multi-node V2X communication.

Therefore, if the light sources of multiple nodes do not overlap within the field of view, it is possible to receive each light source by separating multiple sources through image processing techniques. Figure 6 shows an example of the divided region of interest (ROI) for communication. This divided communication area can be obtained by using various image processing techniques, such as the lane recognition, vehicle recognition, traffic sign recognition under driving traffic environment [10,11]. By dividing the communication area in this way using image processing, we may achieve the simultaneous multiple access communication without inter-channel (inter-node) interferences.

If we divide the node-specific communication area, we may communicate without interference. In this scenario example, we apply the GCM based visual-MIMO to the target color of a traffic light and we may guarantee the required SER performance under worse traffic conditions. The image processing based division of the communication area can also significantly reduce the complexity in the object detection process. This allows a flexible change from time to time to cope with the various situations of the V2X communication.

**Figure 7:**
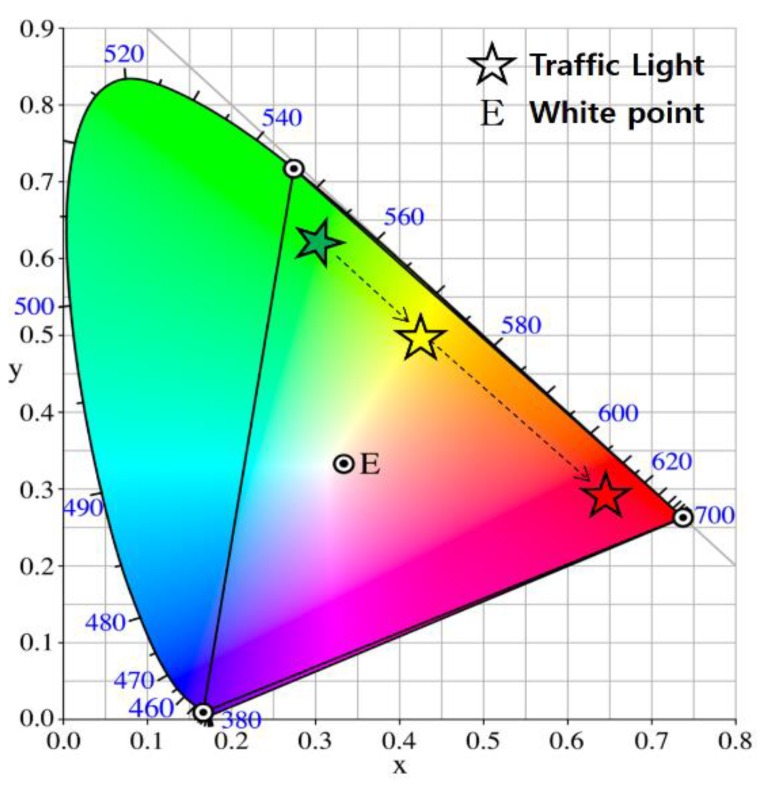
An example of target color transition in a color-space-based constellation diagram.

Figure 7 shows an example of target color transition in a color-space-based constellation diagram, where the target color of traffic light is denoted by a star symbol. Note that ‘E’ denotes the white color in CIE1931 color space. In particular, we consider a case in which the target color of the traffic light changes from green to yellow and from yellow to red. For example, traffic lights can send out their timing and phase information to prevent red light violations and potential accidents and this traffic information is transmitted by symbols (or colors) corresponding to constellation points as explained in Figure 2.

## 3. Results and Discussion

**Figure 8:**
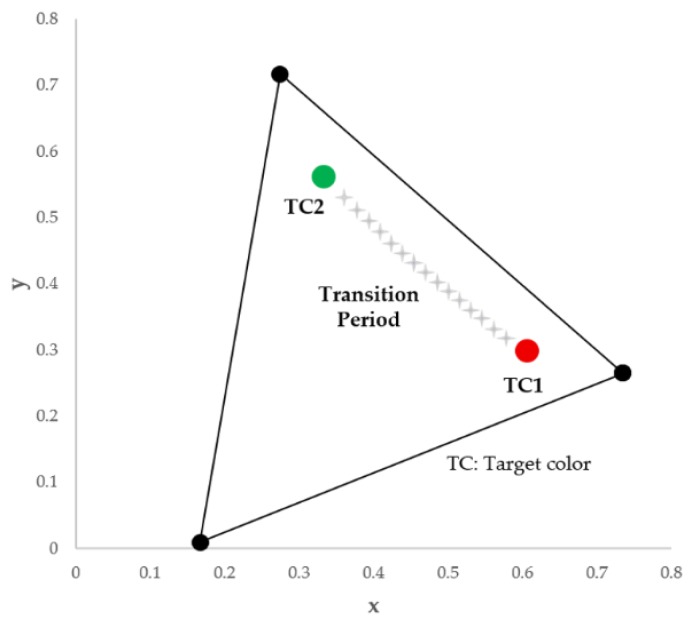
The transition example of a target color of a traffic sign from green to red under GCM based communication between LED and PD.

In the previous GCM based communication between LED and PD, we used a simple average and moving average of the received symbols in order to obtain the target color information at the receiver [7]. So, many errors could occur during the transition and we had to take an error correction coding including interleaving [7]. Figure 8 shows the transition example of a target color of traffic sign from green to red. The error seems like a burst error in RF communication because it appears as a contiguous sequence of error of target color. However, by using visual-MIMO, we can solve this problem successfully since the target color changes with an average of total colors of LED array at every frame.

**Figure 9:**
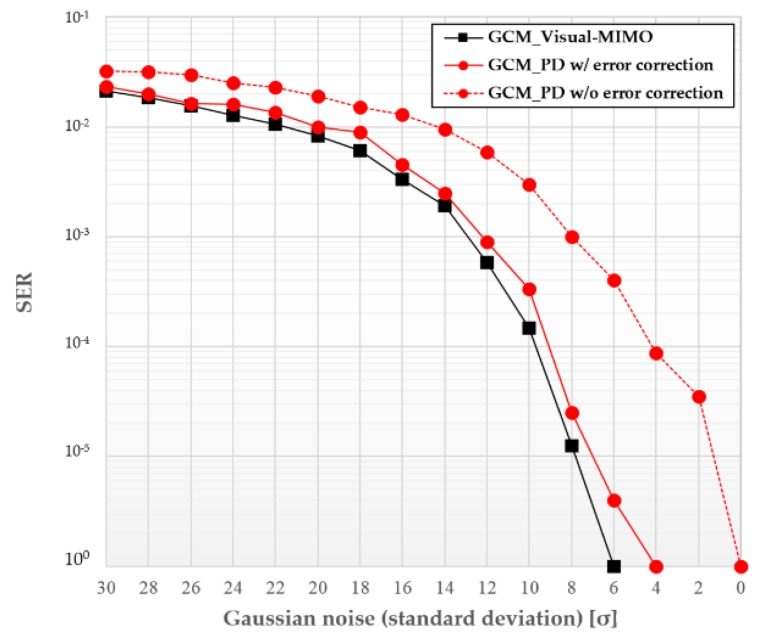
SER performance comparison between visual-MIMO and LED-PD communication under color variation.

Figure 9 shows the superior SER performance of the visual-MIMO scheme compared with LED-PD communication.

**Figure 10:**
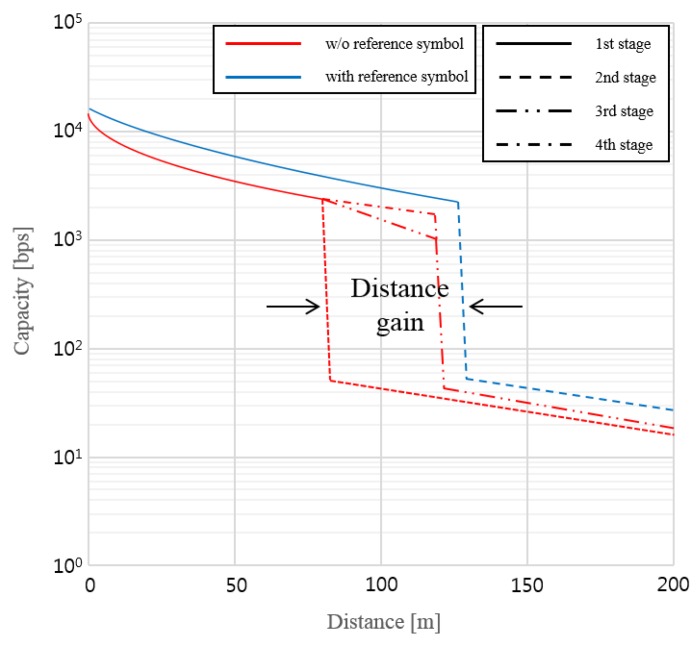
Numerical results of GCM based visual-MIMO (Channel capacity *vs.* Distance).

Figure 10 shows the numerical result of the GCM-based visual-MIMO channel capacity *versus* distance [2]. This result is based on the analytical capacity plots of [1]. In our numerical example, we used the LEA with a size of 16 × 16, 4 symbols, and a frame rate of 30 fps.

If the distance between the source and destination is short enough to guarantee multiplexing transmission (first stage), the capacity is roughly 10 kbps. Then, when the distance has increased and exceeded the limit of the required bit error rate (BER) performance (second stage), there needs to be a transition from the multiplexing mode to the diversity mode. Although we may meet the BER performance requirement, the loss of capacity is unavoidable. However, by relaying the information message to the destination node (third stage), the source node can communicate with the multiplexing mode, providing a capacity comparable to that of the first stage. Moreover, by performing simultaneous transmissions through the relay and on the direct link (fourth stage), we can also obtain an additional SNR gain because of the path-diversity effect.

**Figure 11:**
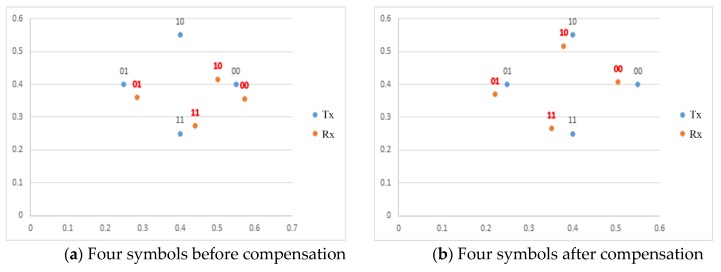
Compensation of channel noise by sending a reference color in the color space of CIE1931.

Most importantly, by sending the reference color (symbol) as part of the networking information, we may achieve improvements in the SER performance because of noise cancellation. In general, effects of channel noise on the transmitted symbols of the LEA in the same frame (or time) are assumed to be almost same. An example of experimental results is shown in Figure 11. We can see that four symbols are represented in the color space of CIE1931. Note that transmitted symbols and received symbols are denoted by blue dot and red dot, respectively. Without using a reference color, as shown in Figure 11a, the probability of symbol error becomes large due to the channel noise. However, if we use a reference color, we may correct the symbol error by compensating the noise. As a reference color, in this experiment, the target color corresponding to the average color of four symbols is used in order not to impair the wanted original color of the lighting. The effect of performance improvement by sending a reference symbol is also shown in Figure 10 by the expected distance gain extending the multiplexing mode.

From Figure 10, we see that the system can achieve large multiplexing gains at short distances and at almost all viewing angles, which implies that it is robust to any misalignment between the transmitter and receiver. It is clear that at large distances, because of the noise effect, the LEDs may not be easily resolved even if the transmitter and receiver are properly aligned; hence, at such distances where multiplexing will fail, we can use diversity to satisfy the required BER performance. Such robustness of the visual-MIMO to any misalignment between the transmitter and receiver is important, especially when applying V2X communication, so, the choice of multiplexing and/or diversity depends largely on the distance between the source and destination node.

## 4. Conclusions

In this paper, we showed the applicability of GCM-based visual-MIMO for V2X. By using the proposed visual-MIMO scheme, while performing seamless communication, we can maintain the original color and brightness in addition to increasing the capacity by using color encoding. First, by performing a numerical example with a scenario, we showed that the choice of multiplexing and/or diversity depends largely on the distance between the source and destination node. Moreover, by performing a simultaneous transmission through the relay and on the direct link, we could obtain the additional SNR gain because of the path-diversity effect. Secondly, by dividing the node-specific communication area, we could achieve the simultaneous multiple access communication without inter-channel (inter-node) interferences. Finally, we demonstrated numerically that improvements in the SER performance through noise compensation could be achieved by sending the reference color (symbol) in the transmitted networking information. This improvement effect was represented by the expected distance gain, which extended the multiplexing mode period.

## Figures and Tables

**Figure S1:**
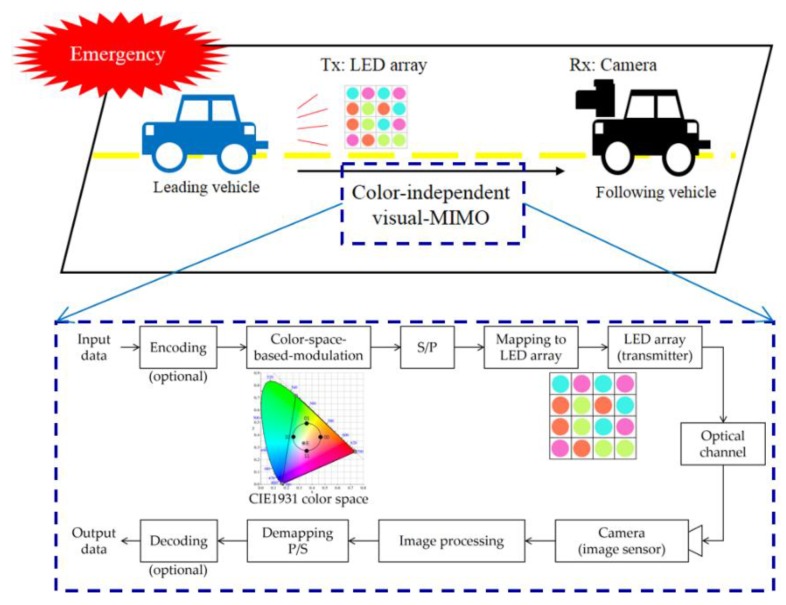
The illustration of V2V communication using color-independent visual-MIMO.

## Supplementary Materials

Figure S1: The illustration of V2V communication using color-independent visual-MIMO.

## Abbreviations

The following abbreviations are used in this manuscript:

BER	bit error rate
CMOS	complementary metal-oxide semiconductor
CSK	color shift keying
GCM	generalized color modulation
ISI	inter-symbol-interference
LEA	light emitting array
LOS	line-of-sight
MIMO	multiple-input multiple-output
PD	photo detector
QAM	quadrature amplitude modulation
RF	radio frequency
ROI	region of interest
SER	symbol error rate
SNR	signal-to-noise ratio
V2V	vehicle-to-vehicle
V2X	vehicle-to-everything
VANETs	vehicular ad hoc networks
VLC	visible light communication
